# Joint recommendations on cost calculation and estimation in paediatric clinical trials

**DOI:** 10.3205/000330

**Published:** 2024-04-24

**Authors:** Gabriele Ahne, Julia Nagel, Axel R. Franz, Antje Neubert, Kristina Schachtrup, Simone Helms, Sebastian Klammt, Matthias Schwab

**Affiliations:** 1 Dr. Margarete Fischer-Bosch-Institute of Clinical Pharmacology (IKP), Stuttgart, Germany; 2 German Network for Paediatric Trials (GermanNetPaeT), Stuttgart, Germany; 3 Paediatric Clinical Study Centre, University Hospital Erlangen, Germany; 4 KKS Network (KKS-Netzwerk e.V.) Berlin, Germany; 5 Centre for Paediatric Clinical Studies (CPCS) University Hospital Tübingen, Germany; 6 Clinical Trials Unit, Medical Centre University of Freiburg, Faculty of Medicine, University of Freiburg, Germany; 7 Department of Pediatrics and Adolescent Medicine, University Hospital Münster, Germany; 8 Department of Clinical Pharmacology, and Pharmacy and Biochemstry, University Tübingen, Germany

**Keywords:** paediatrics, cost calculation, clinical trial

## Abstract

The conduct of clinical trials in paediatrics is essential to improve drug therapy in children. In Europe, paediatric clinical trials have been supported by the European Paediatric Regulation since 2007, but there is still a great need for high-quality clinical trials.

The personnel and time required to conduct clinical trials in accordance with EU Regulations 536/2014 and 745/2017 is considerably higher compared to other studies, such as observational studies. It is important that this additional workload for the trial centre is fully compensated, also taking into account EU state aid rules. In paediatric trials, it is necessary to take into account the special requirements of paediatric and adolescent medicine when calculating the additional costs.

Within the framework of the pan-European paediatric study network c4c/GermanNetPaeT, a working group dealt with specific aspects of cost calculation in order to support paediatric study centres in internal cost calculation as well as in the subsequent preparation of financing requirements for industrial sponsors or public funders.

In several workshops the working group developed a cost calculation template with the content derived from the “Joint recommendations for a total services account as a factor in simplifying contracts” of the Deutsche Hochschulmedizin (DHM, German University Medicine), the Netzwerk der Koordinierungszentren für Klinische Studien (KKS Network, Network of Coordinating Centres for Clinical Trials) and the Verband Forschender Arzneimittelhersteller (vfa, German Association of Research-Based Pharmaceutical Companies).

By estimating the specific time required for measures and investigations as part of a sample study, the background to the increased time required was discussed and a list with aspects to be considered for cost calculation was compiled together with the study centres.

The paediatrics-specific aspects mentioned in detail are intended to increase understanding of the particular problem of higher costs for clinical trials involving children and adolescents and the need for correspondingly appropriate remuneration. This transparent and comprehensible presentation of the higher financial requirements for both the study centres and the financial supporters is intended to promote the high-quality conduct of clinical trials in paediatric study centres in the long term.

## Abstract (de)

Die Durchführung von klinischen Prüfungen in der Pädiatrie ist essentiell für die Verbesserung der Arzneimitteltherapie bei Kindern. In Europa werden pädiatrische klinische Prüfungen seit 2007 durch die European Paediatric Regulation gefördert; trotzdem besteht nach wie vor ein hoher Bedarf an qualitativ hochwertigen klinischen Studien.

Der personelle und zeitliche Aufwand bei der Durchführung von klinischen Prüfungen entsprechend den EU-Verordnungen 536/2014 und 745/2017 ist im Vergleich zu anderen Studien, wie z.B. Beobachtungsstudien, erheblich größer. Es ist wichtig, dass dieser Mehraufwand des Prüfzentrums auch unter Berücksichtigung des EU-Beihilferechts vollkostendeckend vergütet wird. Bei pädiatrischen Studien ist es bei der Kostenermittlung des Mehraufwands notwendig, die besonderen Anforderungen der Kinder- und Jugendmedizin zu berücksichtigen.

Im Rahmen des paneuropäischen pädiatrischen Studiennetzwerks c4c/GermanNetPaeT beschäftigte sich eine Arbeitsgruppe mit spezifischen Aspekten der Kostenkalkulation, um pädiatrische Studienzentren bei der internen Kostenermittlung ebenso wie nachfolgend auch bei der Erstellung des Finanzierungsbedarfes für industrielle Sponsoren oder öffentliche Förderer zu unterstützen.

In Workshops erarbeitete die Arbeitsgruppe ein Kostenkalkulationstemplate, welches sich inhaltlich von den „Gemeinsamen Empfehlungen zur Erstellung einer Gesamtleistungsrechnung“ von Deutscher Hochschulmedizin, dem Netzwerk der Koordinierungszentren für Klinische Studien (KKS-Netzwerk) und dem Verband Forschender Arzneimittelhersteller (vfa) ableitet.

Durch die Abschätzung des konkreten Zeitbedarfs für Maßnahmen und Untersuchungen im Rahmen einer Beispielstudie wurden die Hintergründe des erhöhten Zeitbedarfs erörtert und zusammen mit den Studienzentren eine Liste mit Hinweisen für die Kostenkalkulation erstellt.

Die im Detail benannten Pädiatrie-spezifischen Aspekte sollen das Verständnis für die besondere Problematik höherer Kosten für klinischen Prüfungen mit Kindern und Jugendlichen und die Notwendigkeit einer dementsprechend angemessenen Vergütung erhöhen. Durch diese transparente und nachvollziehbare Darstellung der höheren Finanzbedarfe sowohl für die Studienzentren als auch die finanziellen Unterstützer soll langfristig die qualitativ hochwertige Durchführung von klinischen Prüfungen in pädiatrischen Studienzentren gefördert werden.

## Background

The conduct of clinical trials in paediatrics is essential for improving drug therapy in children. This applies both to off-label medication, i.e. drugs not authorised for children, and to new drugs. In the inpatient sector, off-label prescriptions are used in up to 69% of the cases treated [1]. Many therapies have thus not been systematically evaluated, which means that developmental peculiarities in children that affect pharmacokinetics or pharmacodynamics are sometimes not taken into account. Developmental characteristics concern, for example, age-dependent kidney and liver function and the different development of drug-metabolising enzymes and drug transporters [2]. In addition, there is often a lack of adequate paediatric dosage forms that can be administered to young patients [3]. 

In order to promote the development of medicinal products and therapies for children and to ensure the appropriate authorisation of such medicinal products, the European Paediatric Regulation has been in force since 2007. Although the European Paediatric Regulation has already shown positive effects, there is still a great need to conduct clinical trials as part of the development of medicinal products for children and adolescents, especially for diseases that only affect children [4]. 

There are many reasons for delays in the preparation of clinical trials, including lengthy contract negotiations between study centres and sponsors. In accordance with the recommendation of the German Science and Humanities Council in 2018, the Netzwerk der Koordinierungszentren für Klinische Studien (KKS Network, Network of Coordinating Centres for Clinical Trials), the Deutsche Hochschulmedizin (DHM, German University Medicine) and the Verband Forschender Arzneimittelhersteller (vfa, German Association of Research-Based Pharmaceutical Companies) have drawn up model contract clauses that serve as a guide and starting point for drafting contracts between academic institutions and industrial sponsors. They provide examples of certain recurring contractual provisions in contracts for the conduct of clinical trials, such as confidential information, liability, termination or cancellation of a contract, and publications. These model contract clauses were revised in 2023 and wording on aspects of data protection and the rights to results in the context of clinical trails was included [5].

Before the start of a clinical trial, a study agreement must be concluded, which also regulates the compensation of the study-related additional expenses between the sponsor and the study centre. Compared to other studies, e.g. observational studies, the additional personnel and time required for clinical trials in accordance with EU Regulations 536/2014 [6] and 745/2017 [7] is considerably higher. In accordance with state aid rules, for contract research full cost-covering remuneration must be ensured for the trial centre for all study-related tasks.

In Germany, cost calculation for paediatric studies is carried out on a study-specific basis in the sense of an effort estimation in the individual centres, whereby support is often offered by local clinical study centres at university sites. The joint recommendations for a total services account as a factor in simplifying contracts by German University Medicine, the KKS Network and the vfa are helpful here, especially in order to take into account those activities within the framework of clinical trials that are not usually listed in the study protocol [8].

Paediatric drug studies require additional time and effort compared to studies in adults, which must be taken into account when calculating costs. This time expenditure is often underestimated [9]. Initial ideas from industrial sponsors (initial proposals) regarding remuneration often do not take this specific additional effort into account and therefore often impede a cost-covering realisation of the trial. The corresponding adjustments in the contract negotiations are therefore often very time-consuming. 

## Working group on cost calculation – conect4children – GermanNetPaeT

Conect4children (c4c) is a European network with the aim of promoting the development of new paediatric drugs and, in particular, the conduct of clinical drug trials. As part of the European funding programme “Innovative Medicines Initiative 2 (IMI2)”, a public-private partnership between the European Union and the European pharmaceutical industry, 35 academic and 10 industrial partners are working together in c4c across Europe [10]. GermanNetPaeT is the German national academic contact in the pan-European paediatric study network, within the framework of which the working group “Cost and effort estimation for paediatric clinical trials” was established. Through intensive exchange and the development of joint recommendations on cost for paediatric drug trials, the aim is to create a basis to support the paediatric study centres in cost calculation and thus raise awareness of the special time required to conduct paediatric drug trials among industrial sponsors and public third-party funders. 

As individual study-specific additional costs must be taken into account for each clinical trial, the development of a generally binding cost calculation template was not the aim of this working group. However, statements can be made on general comprehensive aspects of paediatric trials and the resulting costs can be estimated. 

The working group “Cost and effort estimation for paediatric clinical trials” consisted of collaborators from eight academic study centres in Germany and representatives of GermanNetPaeT, who developed a cost calculation template in several workshops (04/2019–02/2022).

## Results

### Cost calculation template for a study centre

The content of the developed cost calculation template is derived from the “Joint recommendations for a total services account as a factor in simplifying contracts” [8]. It is divided into key data (including information on the number of visits, recruitment period, number of patients in the study centre, hourly rates for personnel), activities during study set-up, flowchart of visits (study-specific procedures and examinations), general activities and end-of-study activities. For practical use in the calculation, the document was created in Excel format and the corresponding instructions in PDF format.

The PDF version of the calculation template can be provided by GermanNetPaeT on request.

### Special paediatric features in the cost estimation for a trial centre

Paediatric clinical trials are often characterised by a small number of patients per centre and thus by longer durations compared to studies with adult patients. Longer follow-up periods must also generally be planned. This implies a considerable and continuous additional effort (e.g. training and maintenance of staff, updating the investigator file). Paediatric studies are often dependent on numerous trial centres due to the limited number of patients suitable for the study in one centre. Nevertheless, qualified study personnel must be available at each centre in order to recruit the often small number of eligible children for a drug trial. This particular situation is often underestimated by sponsors, as in clinical trials with adults for diseases with a high prevalence, such as arterial hypertension, type 2 diabetes mellitus, bronchial asthma, or renal insufficiency, a few trial centres are often sufficient to ensure recruitment of the planned number of cases. 

An overview of relevant paediatric particularities in clinical trials is given in Table 1 (Tab. 1).

In order to take account of paediatric particularities for the cost calculation of clinical trials, five study centres of the working group have estimated the time required for selected study-specific requirements or examinations using the example of a selected paediatric study [11], [12] and discussed the background. The results are summarised in Table 2 (Tab. 2). It should be noted that the time requirements mentioned are based on the experience of the participants and do not represent generally binding time estimates.

The sample study has the following design: it is a multicentre, randomised, open-label study with blinded endpoint evaluation to investigate the use of corticosteroids plus intravenous immunoglobulin (IVIG) and aspirin compared to IVIG and aspirin for the prevention of coronary artery aneurysms in children and adolescents with Kawasaki syndrome aged between 30 days and 15 years. The aim of the study is to test whether corticosteroids plus standard treatment lead to a better treatment outcome than standard treatment alone. The study information is taken from the EU Clinical Trial Register, where the study is registered under the EudraCT number 2019-004433-17. 

In order to better justify the time required, Table 3 (Tab. 3) provides a detailed description of the necessary study-specific measures.

## Discussion and conclusion

Clinical trials in paediatrics have a number of particularities that need to be taken into account when estimating costs. Appropriate quality and informative value can only be ensured if the clinical trial is financed to cover the costs. This is also a basic ethical requirement for the trial participants. According to the “Erste Stellungnahme und Empfehlung der Regierungskommission für eine moderne und bedarfsgerechte Krankenhausversorgung“ (first statement and recommendation of the government commission for modern and demand-orientated Hospital Care), in routine care, too, awareness must be created that, compared to adult medicine, the amount of child- and parent-appropriate consultation and care required in paediatrics is significantly higher [13]. This also applies in particular to the provision of information and consent for clinical drug trials.

A realistic estimate of the duration of the study before the start of the trial is essential for a cost-covering calculation. The developed cost calculation template facilitates the planning of the effort for the total duration of the study. Estimating the individual workload for processing of adverse events (e.g. serious adverse events (SAEs)), monitoring visits or audits and the associated costs before the start of the study is often a particular challenge. It therefore seems appropriate and sensible to remunerate these services according to the actual number of services performed in line with a pre-agreed reimbursement.

In summary, the described specific cost aspects of paediatric studies compared to clinical trials in adults are intended to raise awareness of the particular problems and clarify the need for appropriate remuneration for clinical trials in children. This is intended to serve as a basis for argumentation vis-à-vis industrial sponsors and other funders in order to enable appropriate and cost-covering remuneration of clinical paediatric trials. Only with a common understanding of all stakeholders about the higher, but also justified costs of paediatric trials compared to other clinical trials can the high-quality conduct of clinical drug trials in paediatrics and thus evidence-based therapy for children and adolescents in Germany and Europe be ensured in the long term.

## Notes

### Acknowledgements

The “Joint recommendations on cost calculation and estimation in paediatric clinical trials” were developed within a working group of the GermanNetPaeT (German Network for Paediatric Trials) 2019–2022 as part of the pan-European project c4c (conect4children) and are available in German and English. The KKS-Netzwerk e.V. supported the GermanNetPaeT as part of the c4c project. G. Ahne und M. Schwab were in parts supported by the Robert Bosch Stiftung, Stuttgart.

### Working group

The working group consisted of:

- Ahne, G. Dr., IKP Stuttgart, Project Management GermanNetPaeT
- D’Amario, A., CPCS Tübingen
- Franz, A. Prof., CPCS Tübingen
- Grählert, X. Dr., KKS Dresden
- Helms, S., University Hospital Münster
- Klammt, S. PD Dr., KKS-Netzwerk, Berlin
- Köhn, M., ZKS Tübingen
- Lucht, M., KKS-Netzwerk, Berlin
- Maas, S. Dr., CCS Erlangen
- Nagel, J. Dr., KKS-Netzwerk, Berlin
- Neubert, A. Prof., Centre for Paediatric Clinical Trials Erlangen
- Neumann, E., IKP Stuttgart, Project Management GermanNetPaeT
- Schachtrup, K. Dr., ZKS Freiburg
- Schneidewind, A., Hauner iPSC Munich
- Schön, H., ZKS Tübingen
- Schwab, M. Prof., IKP Stuttgart, Head of GermanNetPaeT

### Shared first authorship

Gabriele Ahne and Julia Nagel share first authorship.

### Competing interests

The authors declare that they have no competing interests.

## Figures and Tables

**Table 1:**
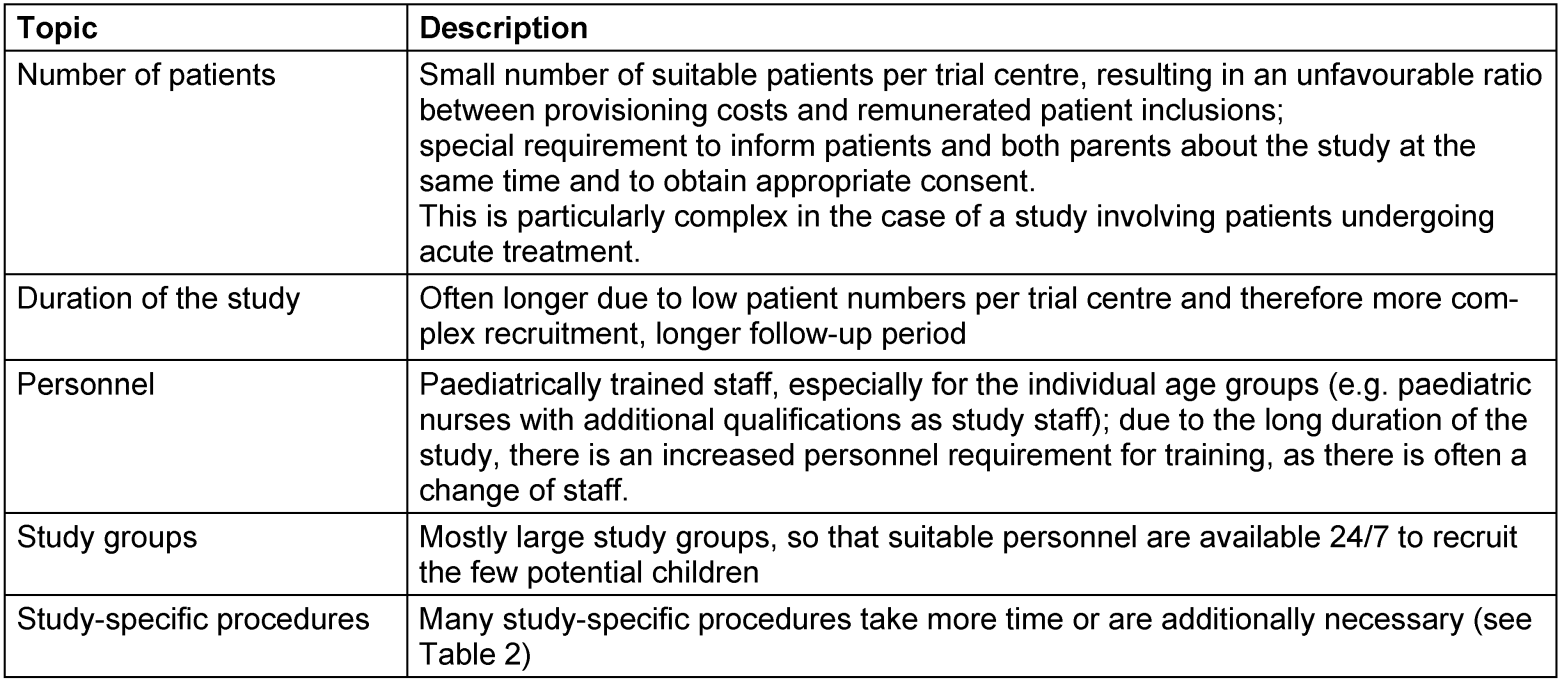
Paediatric particularities in clinical trials

**Table 2:**
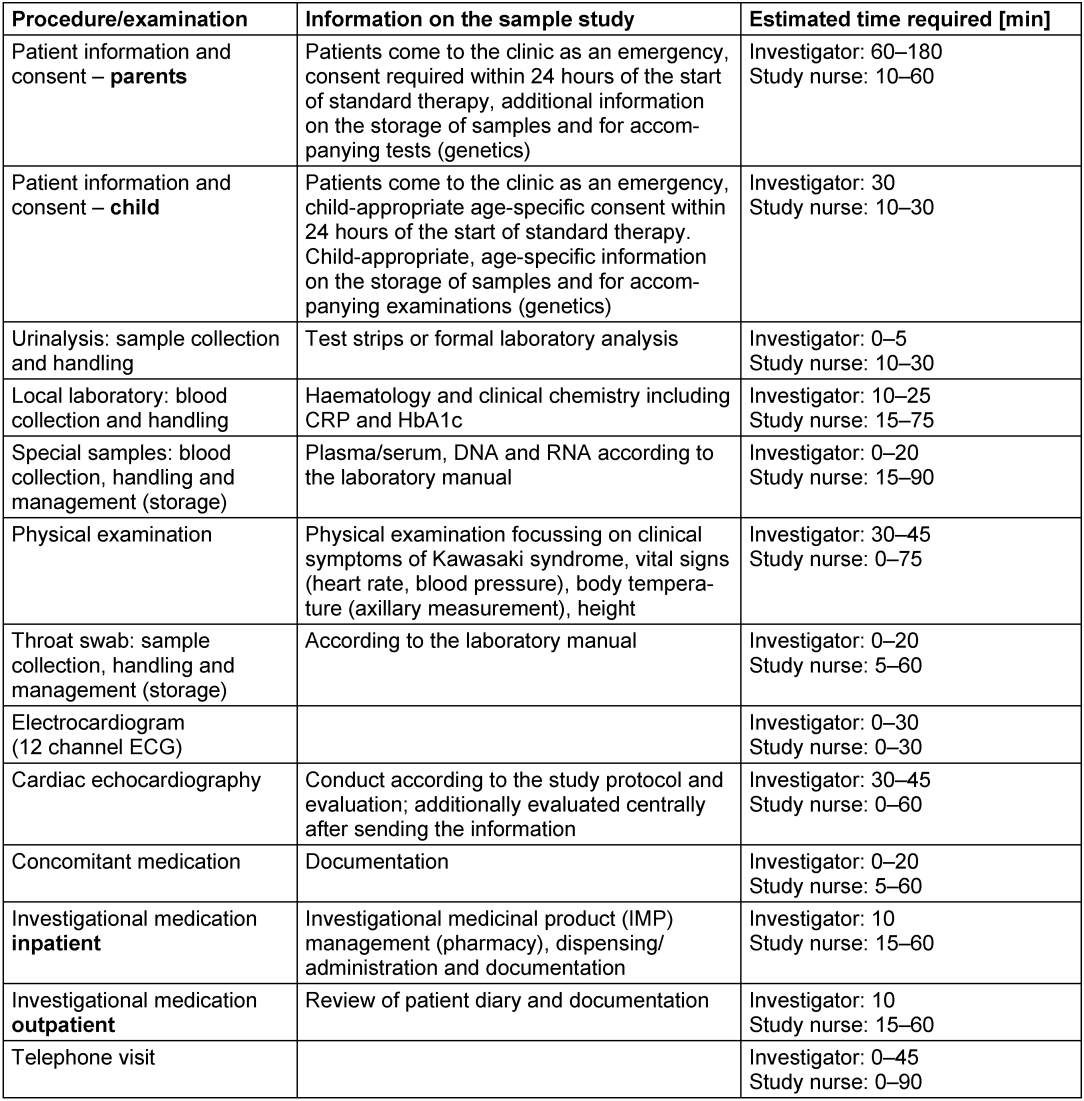
Estimation of the time required for study-specific procedures and examinations in the sample study [11], [12]

**Table 3:**
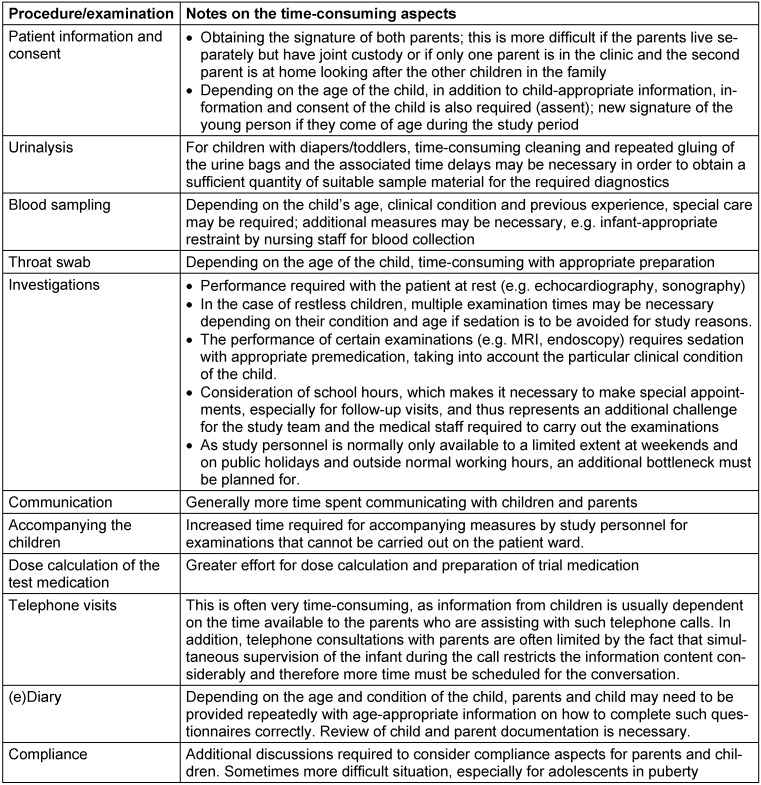
Special paediatric features of study-specific procedures and examinations

## References

[1] Weda M, Hoebert J, Vervloet M, Moltó Puigmarti C, Damen N, Marchange S, Langedijk J, Lisman J, van Dijk L (2017). Study on off-label use of medicinal products in the European Union.

[2] Kearns GL, Abdel-Rahman SM, Alander SW, Blowey DL, Leeder JS, Kauffman RE (2003). Developmental pharmacology--drug disposition, action, and therapy in infants and children. N Engl J Med.

[3] Zahn J, Hoerning A, Trollmann R, Rascher W, Neubert A (2020). Manipulation of Medicinal Products for Oral Administration to Paediatric Patients at a German University Hospital: An Observational Study. Pharmaceutics.

[4] European Commission (2017). State of Paediatric Medicines in the EU: 10 years of the EU Paediatric Regulation. Report from the Commission to the European Parliament and the Council.

[5] KKS-Netzwerk, Verband Forschender Arzneimittelhersteller e.V. (vfa), Deutsche Hochschulmedizin e.V., Bundesverband der Pharmazeutischen Industrie e.V., Bundesverband Medizinischer Auftragsinstitute e.V. (2023). Mustervertragsklauseln für klinische Prüfung mit Arzneimitteln unter Verantwortung eines pharmazeutischen Unternehmens (industrieller Sponsor).

[6] (2014). Verordnung (EU) Nr. 536/2014 des Europäischen Parlaments und des Rates vom 16. April 2014 über klinische Prüfungen mit Humanarzneimitteln und zur Aufhebung der Richtlinie 2001/20/EG. Amtsblatt der Europäischen Union.

[7] (2017). Verordnung (EU) 2017/745 des Europäischen Parlaments und des Rates vom 5. April 2017 über Medizinprodukte, zur Änderung der Richtlinie 2001/83/EG, der Verordnung (EG) Nr. 178/2002 und der Verordnung (EG) Nr. 1223/2009 und zur Aufhebung der Richtlinien 90/385/EWG und 93/42/EWG des Rates. Amtsblatt der Europäischen Union.

[8] Bruns I, Schade-Brittinger C, Wissing F, Ruppert T, Trillsch M (2019). Joint recommendations for a total services account as a factor in simplifying contracts. Ger Med Sci.

[9] Siapkara A, Fracasso C, Egger GF, Rizzari C, Trasorras CS, Athanasiou D, Turner MA, Working Group Membership (2021). Recommendations by the European Network of Paediatric Research at the European Medicines Agency (Enpr-EMA) Working Group on preparedness of clinical trials about paediatric medicines process. Arch Dis Child.

[10] Turner MA, Hildebrand H, Fernandes RM, de Wildt SN, Mahler F, Hankard R, Leary R, Bonifazi F, Nobels P, Cheng K, Attar S, Rossi P, Rocchi F, Claverol J, Nafria B, Giaquinto C (2021). The conect4children (c4c) Consortium: Potential for Improving European Clinical Research into Medicines for Children. Pharmaceut Med.

[11] Eleftheriou D, Moraes YC, Purvis C, Pursell M, Morillas MM, Kahn R, Mossberg M, Kucera F, Tulloh R, Standing JF, Swallow V, McCormack R, Herberg J, Levin M, Wan M, Klein N, Connon R, Walker AS, Brogan P (2023). Multi-centre, randomised, open-label, blinded endpoint assessed, trial of corticosteroids plus intravenous immunoglobulin (IVIG) and aspirin, versus IVIG and aspirin for prevention of coronary artery aneurysms (CAA) in Kawasaki disease (KD): the KD CAA prevention (KD-CAAP) trial protocol. Trials.

[12] conect4children KD – CAAP: Kawasaki Disease Coronary Artery Aneurysm Prevention trial.

[13] Regierungskommission für eine moderne und bedarfsgerechte Krankenhausversorgung (2022). Erste Stellungnahme und Empfehlung der Regierungskommission für eine moderne und bedarfsgerechte Krankenhausversorgung: Empfehlungen der AG Pädiatrie und Geburtshilfe für eine kurzfristige Reform der stationären Vergütung für Pädiatrie, Kinderchirurgie und Geburtshilfe (08.07.2022).

